# Learning experiences and perceived educational value of a blended antenatal course among nursing undergraduates: a qualitative study

**DOI:** 10.3389/fmed.2026.1834908

**Published:** 2026-07-20

**Authors:** Xiaoyan Feng, Xiaochang Yang

**Affiliations:** 1 Department of Obstetrics and Gynecology, The First Affiliated Hospital of Chongqing Medical University, Chongqing, China; 2 Department of Nursing, The First Affiliated Hospital of Chongqing Medical University, Chongqing, China

**Keywords:** antenatal education, blended learning, nursing undergraduates, emotional competence, aesthetic sensibility, qualitative study

## Abstract

**Background:**

To explore the learning experiences and perceptions of nursing undergraduates who participated in antenatal classes within a blended learning environment, and to examine the perceived educational value of an innovative teaching model that integrates rational, emotional, and aesthetic education.

**Methods:**

Eighty-six third-year nursing undergraduates from Chongqing Medical University participated in a blended learning program consisting of online and offline courses at the First Affiliated Hospital’s Antenatal School, labor room observations, and a science communication competition. Following the intervention, semi-structured interviews were conducted with 20 students. A thematic analysis approach informed by grounded theory coding procedures was employed. Interview transcripts were analyzed using NVivo 12 software for coding and theme extraction.

**Results:**

The coding process generated 62 initial codes, 14 axial codes, and 6 selective codes, which were further refined into two overarching themes: Emotional Competence and Aesthetic Sensibility.

**Conclusion:**

Nursing students’ participation in antenatal classes contributed to the development of emotional competence—encompassing interpersonal sensitivity, emotional intelligence, intellectual engagement, and moral sensibility—and also cultivated aesthetic sensibility through aesthetic and humanistic experiences. These findings underscore the perceived educational value of integrating comprehensive quality education into nursing curricula, offering a meaningful pedagogical model for holistic talent development.

## 1 Introduction

Nursing is widely recognized as a demanding profession characterized by high pressure and workload. Beyond mastering solid professional knowledge and skills, nursing professionals require well-developed emotional capabilities and humanistic literacy. While academic curricula predominantly emphasize rational education, societal expectations for emotional quality and aesthetic sensibility are increasingly prominent. Therefore, understanding nursing students’ subjective feeling, aesthetic perception, and creative thinking before clinical placement is of great value. This helps students establish connections with the real world on physiological, psychological, and practical levels.

As proposed by Zhou Haihong, Vice President of the Central Conservatory of Music, individuals possess three core “qualities”: Rational Quality (Truth-seeking, representing cognitive ability), Emotional Quality (Goodness-seeking, representing affiliative ability, the foundation for interaction, friendship, and kinship), and Aesthetic Sensibility (Beauty-seeking, representing experiential ability, essential for appreciating and creating art and culture, and experiencing life meaningfully) (1). Emotional competence and aesthetic sensibility are concepts relative to rational quality, denoting an individual’s direct feeling and perception capacity toward the goodness and beauty inherent in nature, society, life, and the environment. Emotional competence refers to relatively stable, fundamental, and developmentally appropriate positive emotional-psychological characteristics formed through the interaction of genetics, environment, and practice during the university stage. It primarily pursues morality, representing affiliative ability (2). Aesthetic sensibility encompasses an individual’s direct sensory and perceptual abilities regarding nature, society, life, and the environment, including sensory cognition, sensory intelligence, and receptive capacity. It relates to human aesthetics and the pursuit of a favorable environment. In this sense, it constitutes a unique quality enabling humans to beautify their living environment and themselves, serving as the fundamental drive for artistic creation (3).

Historically, nursing education in China has been perceived as lacking sufficient humanistic depth. Students often choose the profession based on employment prospects rather than a profound understanding of the field, leading to confusion regarding professional values (4). Cultivating nursing students’ respect for life and commitment to safeguarding maternal and infant health from emotional and aesthetic perspectives holds significant benefits. To address this gap, this qualitative study focused on the “Maternal and Newborn Care” module within the Obstetric and Gynecological Nursing course. The teaching design, centered on students, established a social platform connecting students, pregnant women, families, and teachers, aiming to educate through teaching and environmental influence (5). This study explored students’ emotional and aesthetic experiences to gain insights into the perceived educational value of fostering emotional competence and aesthetic sensibility. The findings are reported below.

## 2 Methods

### 2.1 Participants

Purposive sampling was employed. From 86 third-year nursing undergraduates participating in the Antenatal School program of the First Affiliated Hospital of Chongqing Medical University between March and May 2024, 20 were selected for interviews. Inclusion criteria were: completion of all online and offline antenatal school courses, on-site observations, and science communication activities; voluntary participation; and willingness to express themselves fully. The study adhered to the principles of voluntariness and confidentiality, with informed consent obtained from all participants. Sampling continued until data saturation was reached, with no new themes emerging. Participants were 20 female nursing students (mean age 21.00 ± 0.66 years), coded as N1 to N20. Nineteen interviews were conducted face-to-face, and one was conducted by phone. All participants engaged in the science communication innovation competition. Ethical approval was granted by the Medical Research Ethics Committee of the First Affiliated Hospital of Chongqing Medical University (Ethics No: 2025-239-01).

### 2.2 Course implementation

**Figure 1:**
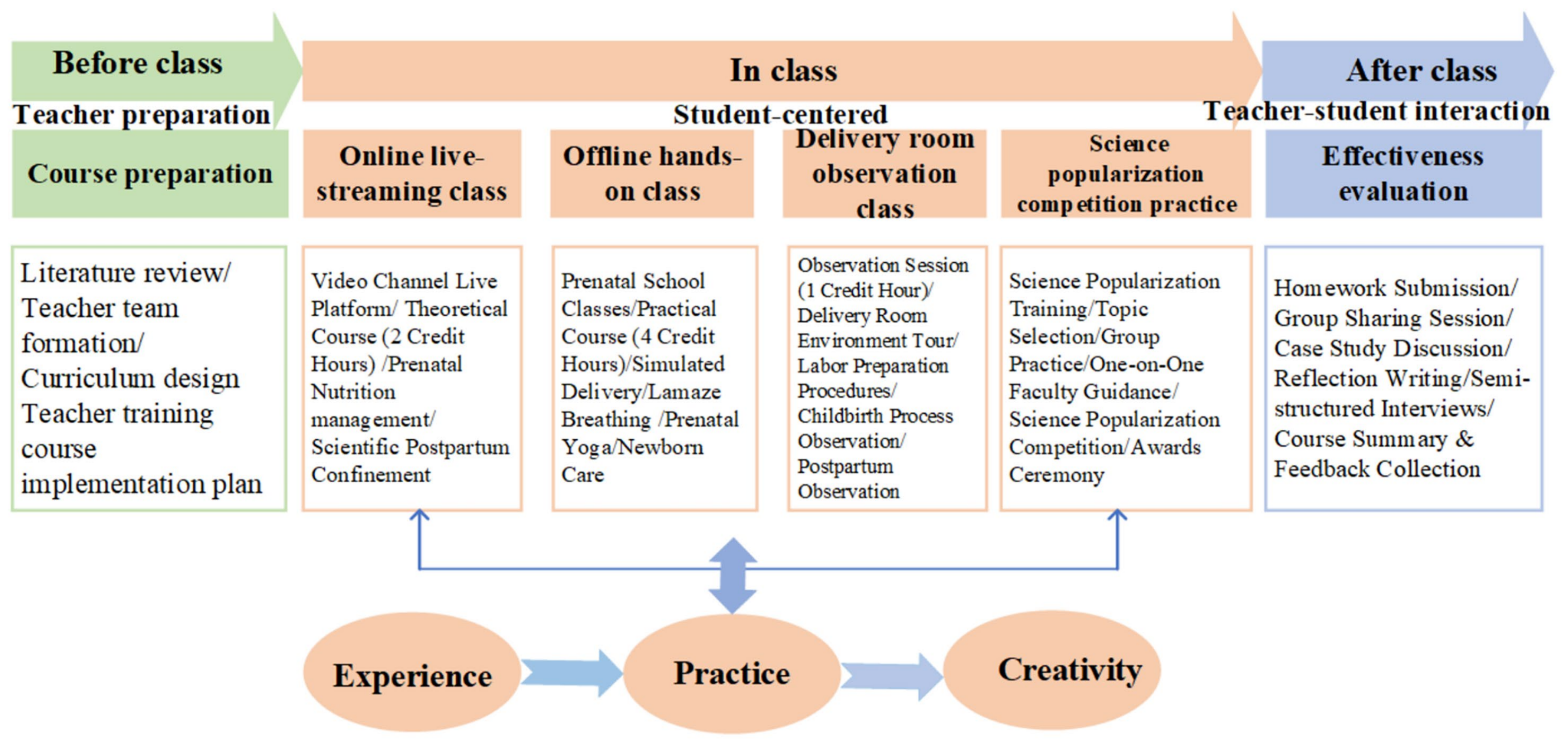
Overall design of the integrated four-component teaching pattern for obstetrics and gynecology nursing students.

As shown in Figure 1, the teaching pattern consisted of four integrated components: online courses, offline courses, labor room observations, and a science communication competition.

(1) Online Courses (2 credit hours): Faculty organized two live-streamed sessions through the Obstetrics Department Video Channel of the First Affiliated Hospital. Content included prenatal nutrition and weight management, as well as scientific postpartum care, followed by expert Q&A. Post-session activities involved case analyses, group discussions, reflective journals, and plotting BMI growth curves for gestational weight management.
(2) Offline Courses (4 credit hours): Held at the Antenatal School, sessions covered simulated delivery, Lamaze breathing techniques, prenatal yoga, WAFF training, and newborn care.
(3) Practical Activities (2 credit hours): During guided labor room visits, students observed the labor and delivery process in batches.
(4) Science Communication Competition and Award Ceremony: Post-course, faculty provided science communication training. Students worked in groups to create popular science materials under faculty mentorship. Twenty-six entries were submitted to the New Media Nursing Science Communication Works competition organized by the Obstetrics Committee of the Chinese Nursing Association in 2024. The award ceremony involved pregnant women, families, obstetric staff, faculty, and students. Activities included poetry recitation, birth dance performances, sharing of pregnancy and delivery experiences by mothers, and a concluding circle singing “Grateful Heart” to offer blessings.

### 2.3 Research methods

#### 2.3.1 Interview guide development

**Figure 2:**
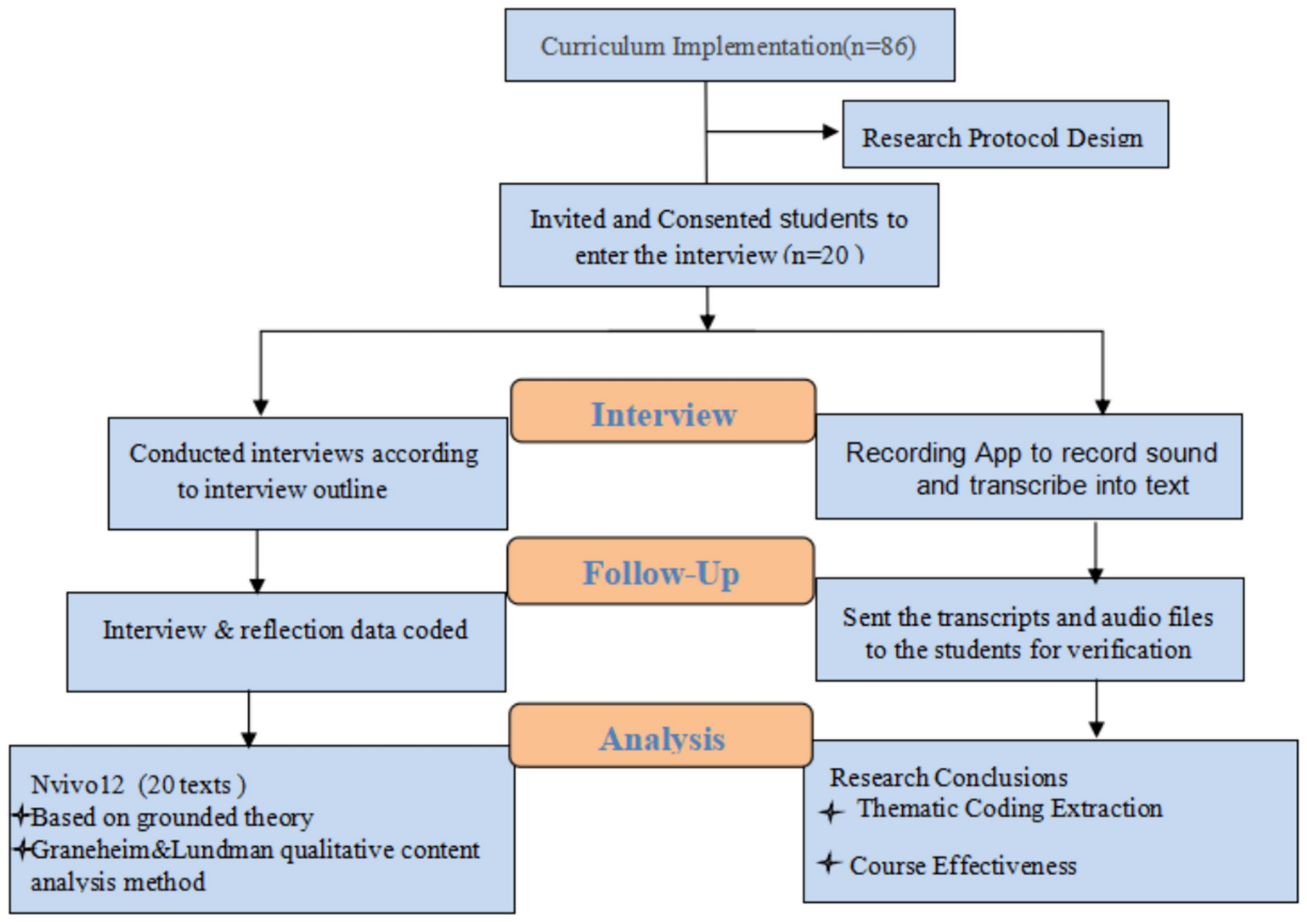
Overview of the research design.

An initial interview guide was drafted based on literature review and research objectives. It was refined following consultations with one qualitative research expert and two nursing education experts, and adjusted based on feedback from pilot interviews. The final semi-structured interview guide is presented in Table 1. This qualitative study is reported in accordance with the COREQ checklist. The research pathway is shown in Figure 2.

**Table 1:** Semi-structured interview outline.

No.	Questions
1	How do you feel about taking prenatal classes?
2	How do you perceive the experience of integrating maternal education classes into the instructional curriculum?
3	Compared with traditional teaching methods, have you experienced clear academic benefits from this blended learning model?
4	What did you know about the delivery process before? And how did your understanding change after the lessons?
5	Did the science communication competition and award ceremony influence your academic motivation or career plans?

#### 2.3.2 Data collection via semi-structured interviews

As described in the Participants section, 20 of the 86 students were purposively selected for interviews based on data saturation. Researchers explained the study process and significance and obtained consent for audio recording, assuring confidentiality. Interviews began with an ice-breaker question (“How did you feel about the course?”) to establish rapport, followed by questions guided by the interview guide. Researchers employed active listening and appropriate prompts to encourage open expression. The 30-min interviews were conducted face-to-face or by phone in the Antenatal School lounge and were audio-recorded. Field notes were taken during and immediately after each interview to record non-verbal cues, contextual observations, and researcher reflections.

All interviews were conducted by the lead researcher, Xiaoyan Feng, a female midwife and faculty member at a medical university with a Master’s degree in Psychology. She has received formal training in qualitative methods and is proficient in interviewing techniques such as probing, prompting, and demonstrating empathy, which helped ensure research quality.

#### 2.3.3 Data analysis

Recordings were transcribed verbatim. Transcripts and recordings were sent to participants for verification and correction to ensure accuracy. Data collection and analysis occurred concurrently. NVivo 12 (6) was used for data management and analysis. A thematic analysis approach informed by grounded theory coding procedures (7, 8) was employed. The analysis followed these steps:

First, open coding (initial coding). Two researchers independently performed open coding on a subset of transcripts, conducting line-by-line coding of the interview data, identifying meaningful segments of text, and generating initial codes. Discrepancies were resolved through discussion, sometimes involving a third researcher, to establish consensus and develop a coding manual.

Second, axial coding. Following the coding manual, the remaining transcripts were coded. Similar codes were then grouped into categories and subcategories based on conceptual relationships.

Third, selective coding. Categories were further synthesized to identify overarching themes or dimensions that captured participants’ core experiences and perceptions.

Preliminary findings, including the identified themes and representative quotes, were shared with participants for member checking. They confirmed that the interpretations accurately reflected their experiences. Returning the interview transcripts and coding results to the participants for validation helped ensure data credibility.

#### 2.3.4 Reflexivity

The lead researcher was a nurse at the affiliated hospital and a faculty member at the medical university. To minimize potential power imbalance and social desirability bias, the following measures were taken. First, the researcher was not involved in any teaching or evaluative activities related to the course. Second, the researcher did not know any of the participants prior to the study, and all interviews were conducted only after the completion of the teaching activities. Participants were explicitly informed that their responses would have no impact on their academic performance or grades, and that there were no “right” or “wrong” answers. All interviews were conducted in a neutral, private setting (the Antenatal School lounge) separate from the formal learning environment. Participants were assured of the confidentiality of their responses and were encouraged to speak candidly. These measures were taken to facilitate open and honest expression and to reduce the influence of perceived authority relationships on data quality.

## 3 Results

Twenty nursing students participated in interviews, generating approximately 36,960 words of transcript data, supplemented by about 22,340 words from post-course reflective journals. Progressive coding resulted in 532 raw nodes, consolidated into 62 initial codes, 14 axial codes, and 6 selective codes. Through constant comparison and reorganization, establishing logical relationships between codes, two overarching themes emerged: Emotional Competence and Aesthetic Sensibility (node distribution detailed in Table 2; See Table 3 for representative quotes from the interviewees).

**Table 2:** Three-level coding items, core thematic framework, and node distribution.

Initial coding	Axial coding	Selective coding	Core theme
Showing care (4), getting along well (4), couple’s bond (12), warm friendship (6), listening to students (2), supportive chats (8)	Perceived caring (4), closeness (16), cooperation (16)	Interpersonal sensitivity (36)	Emotional competence (362)
Not easy (14), happy (8), really looking forward to (19), inner strength (5), moral courage (5), tough grind (15), pure happiness (26), excited (2), really got to me (7), relaxing (13), moved (5), curious (2), surprised (2), worried / scared (4), confused (1), nervous (3)	Understanding others (26), expressing self (39)	Emotional intelligence (131)	
Inspired (2), helped me understand better(40), broadened my knowledge (2), learned more about the field (11), hands-on practice (23), got my priorities straight about studying (3), interest in learning (6), wanting to learn (7), learn with peace of mind (4), serve others better (14), achievement / pride (8), encouragement (3)	Senses of confidence (44), professionalism (34), enjoyment of learning (20), achievement (25)	Intellectual engagement (123)	
Have empathy (3), pay closer attention to care (5), do science education carefully (3), ground science education in reality (6), actual needs(37), mind sets are shifting (16), today’s parents (2)	Senses of responsibility (8), public service (64)	Moral sensibility (72)	
Pure magic (2), unforgettable (24), fresh and exciting (24), totally worth it (4), fascinating (16), hands-on (3), slow-paced (2), glorious (3), keep it real (20), truly extraordinary (11), deeply precious (3), heart warming (6), utterly magical (6), pure miracle (2), life (12)	Sensuous aesthetics (98), life experience (40)	Aesthetic experience (138)	Aesthetic sensibility (170)
Sacred duty (4), love (13), gentle touch (1), heartfelt relief (2), humbled admiration (11), human connection (1)	Humanistic care (32)	Humanistic experience (32)	

The number in parentheses indicates the frequency of the item, “Node count” refers to the frequency of occurrence of extracted terms during coding in Nvivo 12 software.

**Table 3:** Definitions and typical sentences of selective coding.

Selective coding	Axial coding	Definition	Typical sentences
Interpersonal sensitivity	Perceived caring	Refers to an individual’s internal experiences during interactions and social activities with others	I can really feel how all the expectant moms are carefully preparing for their babies, and I feel their love
	Closeness		The teachers are really approachable and kind; The moms-to-be feel like friends
	Cooperation		And I see how important it is for the doctors and nurses team and the moms-to-be to work together – it’s a real team effort
Emotional intelligence	Understanding others	The kind of intelligence demonstrated in handling emotions or feelings	I witnessed boundless love and selfless dedication, which gave me a deeper understanding and respect for motherhood
	Expressing self		This course offered many opportunities to interact with the expectant mothers
Intellectual engagement	Senses of confidence	Refers to an individual’s internal experiences when evaluating cognitive activities and achievements	Understanding the mothers’ true feelings allows us to serve them better
	Professionalism		As professionals staff, we provide them with science-based knowledge
	Enjoyment of learning		I found that this format really motivated me to take a more active role in my own learning
	achievement		Feeling the baby’s back during palpation gave me an incredible sense of achievement
Moral sensibility	Senses of responsibility	Refers to an individual’s internal experiences when evaluating their own and others’ actions against social moral norms	I’ve truly felt how important the prenatal education and emotional support provided by the obstetrics department are
	Public service		Medical success is not just about the operating room; sharing health knowledge is also incredibly significant
Aesthetic experience	Sensuous aesthetics	A profound sense of connection and insight into life’s meaning, sparked by beauty and deepened through truly experiencing it(short for)	Starting the session by singing ‘Grateful Heart’ filled me with gratitude and a sense of life’s miracle and boundless love
	Life experience		What left a deep impression was that final poem, brimming with so much love
Humanistic experience	Humanistic care	A human-centered way of making value judgments, grounded in care and ethical	Clinical work is demanding, but being here taught me the importance of approaching care with compassion

### 3.1 Theme one: emotional competence

This theme encompassed 362 nodes, comprising four axial codes: Interpersonal Sensitivity, Emotional Intelligence, Intellectual Engagement, and Moral Sensibility.

Interpersonal Sensitivity: Refers to an individual’s internal experiences during interactions and social activities with others. This axial code included senses of caring, closeness, and cooperation. Course interactions fostered harmony and intimacy between couples (expectant parents), and cooperation, mutual respect, and encouragement between couples, student-teacher pairs, and expectant parents and students. For instance, regarding interactions with expectant mothers, N10 stated: “During clinical practicum it’s a service relationship between nurse and patient, but at the Antenatal School it felt like friends with the moms.” Another student shared: “When doing a simple fetal heart check, I felt unprofessional and a bit lost, afraid I might be clumsy and hurt the baby. The mom’s ‘It’s okay’ meant so much encouragement.” Students also perceived affection between expectant parents (N9: “I felt they had a very good relationship, a warm emotional atmosphere”). Teacher-student interactions were also noted (N18: “This approach felt very respectful of student needs”).

Emotional Intelligence: Refers to the kind of intelligence demonstrated in handling emotions or feelings. With 131 nodes, this was the largest axial code under Emotional Competence. It comprised two codes: Understanding Others (92 nodes) and Expressing Self (39 nodes). Witnessing pregnant women’s behaviors and expressions in class fostered empathy for the “difficulty” and “hard work” of pregnancy; students sensed the “expectation, ““joy, “and “happiness” associated with new life, alongside feelings of the mothers’ “bravery” and “strength” (N5: “They could persist with such difficult yoga poses! Mothers are truly great!”). These experiences naturally evoked and facilitated emotional expression in students, including feelings of relaxation, surprise, and being moved, alongside curiosity, worry, confusion, and excitement about pregnancy and life (“This learning style felt different from school, very relaxing,” “I felt really surprised by this participatory format,” “Listening to the fetal heartbeat for the first time was nerve-wracking but also exciting”).

Intellectual Engagement: Refers to an individual’s internal experiences when evaluating cognitive activities and achievements. With 123 nodes, this was the second-largest axial code. It included senses of confidence, professionalism, enjoyment of learning, and achievement (professional efficacy). The course model facilitated knowledge consolidation and expansion, enhancing students’ professional “confidence” (N9: “It made me recall key points,” “Learned a lot beyond the textbook, like hearing about the three pelvic joints for the first time”). It also helped students identify gaps in their knowledge (“discovered some gaps in my knowledge”). Compared to traditional lectures, students found the knowledge “very detailed and close to real life, whereas lectures are more macro-level.” Contrasted with hospital practicum (“too serious an atmosphere”), students felt they could “learn without worrying about disputes.” Positioned between traditional theory and clinical placement, the course aided the transition from school to society, theory to practice, and exam focus to application, bridging the development of a “professional” perspective (N1: “gave me a preliminary impression of skills and operations”). It enhanced learning motivation and interest, fostering an “enjoyment of learning” and aligning learning goals (Students: “realized studying this subject is not just for exams, it extends to our lives”). Students experienced “achievement” through practice (“I successfully performed fetal heart monitoring on a mom, located the fetal back, and heard the heartbeat clearly – huge sense of achievement”), reporting the approach “gave me more interest and clearer direction in subsequent learning, “felt “encouraged, “and expressed “pride” in nursing (N7: “I will definitely be able to serve them better”). The course stimulated career aspiration (N13: “It made me a bit interested in working in obstetrics”) and professional identity (N17: “Nurses are really useful”).

Moral Sensibility: Refers to an individual’s internal experiences when evaluating their own and others’ actions against social moral norms. This axial code (98 nodes) included senses of responsibility and public service. Students recognized, both theoretically and emotionally, the need for “more meticulous care” and “empathy” (N18: “We need to see things from their perspective and have empathy”), igniting a sense of “responsibility.” They understood the importance of diverse roles (“Medical success is not just in the operating room; health knowledge popularization is also crucial”). The course allowed them to “understand the most needed knowledge from expectant parents’ perspective, different from a medical student’s, “observing “changing attitudes in the new generation of parents” and realizing “science popularization needs to be detailed and practical.” Engaging in “popularization” was seen as meeting a “real need, “helping students recognize nursing extends beyond hospitals into broader contexts, fostering correct professional awareness and enhancing humanistic care in their professional demeanor.

### 3.2 Theme two: aesthetic sensibility

This theme comprised 170 nodes, encompassing two axial codes: Aesthetic Experience and Humanistic Experience.

Aesthetic Experience: Refers to the state where, based on aesthetic intuition, individuals mobilize their recreative imagination and associative faculties, stir rich emotions, immerse themselves in the artistic work, attain spiritual aesthetic pleasure, and achieve transcendence and life insights through perceptual engagement with the aesthetic object. The course engaged students’ senses, evoking evaluative feelings described as wonderful, unforgettable, novel, necessary, interesting, concrete, slow-paced, beautiful, and authentic (e.g., “I understood the preciousness and wonder of life’s creation!”). Elements like poetry, music, and blessings encouraged appreciation of life from an artistic perspective (“The opening chorus ‘Grateful Heart’ filled me with gratitude, feeling the miracle of life and boundless love”). The course “created a warm and serene atmosphere, “allowing students to feel “how magical life is” and “deepen their appreciation of maternal greatness.”

Humanistic Experience: Refers to the value judgment and moral sentiment centered on human dignity. Students experienced profound humanistic feelings such as love, responsibility, reverence, admiration, gentleness, comfort, and human warmth. Expressions included: “We conveyed love and care to life, ““felt a strong sense of responsibility toward them, ““Learned in clinic it’s too busy; here I learned the importance of a loving attitude, ““my reverence for expectant mothers deepened, ““The teacher assisting delivery was so gentle in her movements and speech, ““Teachers patiently answered moms’ questions in class, ““Whether in class or visiting the labor room, the teachers felt so full of human warmth.”

A word cloud generated from interview transcripts and reflective journals highlighted “Life” as the most frequent term, followed by emotionally evocative words like happiness, expectation, greatness, real, profound, magical, moved, strength, authentic, warmth. Words reflecting cognitive perceptions included responsibility, earnestness, importance, and help.

## 4 Discussion

### 4.1 Perceived development of emotional competence through antenatal class blended learning participation

The findings of this qualitative study reveal that nursing students, after participating in the Antenatal School program, perceived growth in multiple dimensions of Emotional Competence, including Interpersonal Sensitivity (caring, closeness, cooperation), Emotional Intelligence (understanding others, expressing self), Intellectual Engagement (confidence, achievement, enjoyment of learning, professionalism), and Moral Sensibility (responsibility, public service). Participants’ accounts suggest that this educational innovation supports the goals of university-level emotional education by promoting students’ multi-faceted development across learning, living, conduct, and social interaction, and fostering an integration of cognitive, social, and physiological dimensions.

Research by Lu Jiamei et al. indicates that adolescent emotional competence structures involve two levels, six categories, and 29 specific emotions, categorized as ontological (including moral, intellectual, interpersonal, life, and aesthetic emotions) and operational (emotional intelligence) levels, exhibiting distinct age characteristics and educational directionality. Positive interpersonal relationships significantly influence university students’ ontological emotional competence; higher student-rated teacher-student relationship scores correlate with higher overall emotional competence scores. Emotional competence scores increase with the proportion of teachers employing “emotionally engaged teaching, “suggesting harmonious teacher-student relationships elevate emotional competence levels (9). Lu Jiamei et al.’s study on university students’ interpersonal emotions found senses of cooperation and closeness scored lowest. Deficits in cooperative cognition and social skills among adolescents contribute to low cooperation scores (10). Overall, university students’ self-efficacy in interpersonal communication is relatively weak, particularly regarding emotional control and self-worth efficacy. Current students lack confidence in managing emotions during interactions, are prone to negative emotions, and often lack positive self-affirmation experiences (11).

Nursing “core competence” extends beyond theoretical knowledge, clinical skills, observation acuity, and emergency response to encompass strong communication skills, empathy, and the capacity for humanistic care (12). In this study, nursing students described experiencing caring, closeness, and cooperation through their interactions within the Antenatal School setting. They reported that receiving respect and encouragement from teachers and interacting with pregnant women “like friends” fostered a learning atmosphere based on interpersonal connection, which in turn stimulated enjoyment of learning and confidence.

Research indicates a moderate positive correlation between emotional intelligence and career decision-making self-efficacy, with “managing one’s own emotions” and “perceiving emotions” contributing most significantly (13). These influence self-evaluation, information gathering, and goal selection during career decisions. Higher emotional perception ability enables individuals to better recognize and regulate their emotions, select career information more effectively, build confidence in career choices, and conduct more objective self-assessments. Cultivating critical thinking skills in nursing students from the outset prepares them for emotional engagement in clinical practice (14). Simulated clinical learning environments simultaneously promote emotional intelligence and problem-solving skills, enhancing learning satisfaction, with strong interrelationships between these factors (15). Developing nurses’ clinical communication skills—listening, responding effectively, and conveying information to patients and families—is key to patient-centered care. The empathy-altruism hypothesis suggests individuals with higher empathy levels exhibit more helping behaviors; nursing students with high empathy are more likely to detect patient needs and respond appropriately from the patient’s perspective (16). In this study, participants reported that close contact with pregnant women, simulated birth experiences, and observing real births allowed them to authentically perceive life and witness its challenges, fostering empathy for mothers-to-be. This finding suggests such experiences may enhance students’ sensitivity to patient needs in future nursing practice and promote their empathy and emotional self-regulation abilities.

Participants expressed that when nursing students feel satisfied with their learning process, they enter clinical practice with greater confidence and energy (17). Authentic learning, motivation, resilience, support, and collaboration are key factors influencing nursing students’ learning satisfaction. Students value clinically focused courses over purely theoretical ones, finding they help develop professional identity. Factors like meeting learning expectations, duration of experiences, relational aspects (comfort, sense of community), and faculty use of simulation/contemporary teaching technologies impact satisfaction. Prior research identifies self-motivation and personal achievement as primary drivers. Positive self-worth and a “can-do” attitude directly influence well-being and satisfaction. Providing a caring clinical learning environment and positive cooperative relationships enhances students’ adaptability and coping skills when facing challenges (18). Post-intervention interviews revealed that real-world experiential learning provided participants with a more comprehensive understanding of childbirth knowledge and deeper insight into the physical and emotional pain and needs of laboring women. Students reported that the course dispelled childbirth stereotypes, fostered scientific understanding, transformed fear into positive coping, and increased their willingness to pursue obstetric nursing careers. Participants described gaining confidence, achievement, enjoyment of learning, and professionalism through Intellectual Engagement. Immersive experiential learning appeared to stimulate interest and autonomy, guiding students to absorb knowledge and values subtly through contextual immersion (19, 20). Value formation and stability are closely linked to emotions arising from need satisfaction. Bloom’s affective domain taxonomy suggests that sustained emotional development leads to corresponding value formation.

Moral sensibility develops based on individual experiential feelings, manifests as the cultivation of emotional attitudes, and evolves through the mutual influence of moral emotion and cognition (21). Transforming moral heteronomy into moral autonomy is essential for genuine student self-moral education (22). Contemporary challenges among university students include lack of integrity, self-interest tendencies, diminished responsibility, and online moral lapses. In this study, “life” emerged as the most resonant term for students, evoking feelings such as happiness, expectation, and greatness, which in turn fostered cognitive realizations like “great responsibility” and “importance of doctor-patient collaboration.” Feelings of being moved, empowered, authenticity, and warmth contributed to professional “achievement.” Witnessing the real needs of new parents led to the understanding that “science popularization must be practical and necessary.” Participation in creating science popularization materials fostered a sense of public service and altruism (“serving the community”). Authentic interactions with pregnant women, families, and teachers allowed students to experience goodness and beauty in human relationships, igniting professional identity and a sense of responsibility for nursing life, helping solidify a firm professional ethos and life-first consciousness cognitively and emotionally.

Thus, participants’ accounts suggest that this “Third Classroom” model—integrating the Antenatal School—holds unique perceived value in supporting knowledge acquisition and enhancing students’ emotional competence.

### 4.2 Perceived cultivation of humanistic care through aesthetic sensibility education

Students reported experiencing both aesthetic and humanistic care dimensions within this experiential course. Aesthetic sensibility is intrinsically linked to humanistic qualities like emotion, interests, and aesthetic awareness. Sensibility (encompassing sensation and imagination) forms the preliminary cognitive foundation for intellect and reason, essential for human understanding of the world (3). Intellect uses this foundation to transcend experience and grasp the essence of things; reason then validates this existence. Related concepts include “humanistic literacy,” involving emotional experiences and evaluative standards of beauty/ugliness and good/evil, and “aesthetic cognition,” emphasizing the mobilization of spiritual perception through subjective experience (23).

Deficits in students’ aesthetic sensibility education stem from three main causes: lack of aesthetic environments due to rigid teaching models; educational imbalance from over-emphasis on science, rationality, and core focus on logical thinking and “double-base” (basic knowledge/skills) training; and overly specialized art education (24). Universities, as key educational platforms, can enhance aesthetic sensibility through managerial, instructional, and environmental education (1). Higher education should guide students’ internal “feeling, “fostering authentic perceptual habits. Learners connect self-experience with knowledge acquisition by engaging with the real world. Aesthetic education should enhance students’ ability to discern beauty in the objective world, learning to perceive and express it. Curriculum design should integrate traditional methods with advanced educational technology, incorporating literature, art, film, etc., into courses to cultivate aesthetic sensibility through immersive experiences. Teachers should adopt student-centered approaches, respecting emotional needs and using emotional education to activate their inner world, fostering original thinking (25).

Within nursing education, models integrating aesthetic education into professional courses are emerging. Aesthetic education immerses students in aesthetically rich environments, gradually honing their aesthetic appreciation through participation, interaction, and expression. This deepens understanding of the nursing profession, enhances engagement and active practice, improves knowledge retention and teaching effectiveness, fosters professional passion, and cultivates the capacity to enact “beautiful” nursing practice (26, 27). Applying knowledge and skills in specific contexts helps students establish positive professional beliefs and identity. The clinical learning phase is critical for midwifery interns’ role transition. Their experiences and perceptions of the midwife role directly influence their attitude, career choice, and development (28). Academic education should guide students to recognize the dual nature of the role, emphasizing positive reinforcement to inspire professional ethics and resilience. Clinical instructors should model professionalism, prioritize effective communication with students, show care, express empathy, and attend to their physical and mental needs.

This study found that students reported enhanced professional efficacy and perceived “competence” in terms of career aspiration, identity, and achievement. Utilizing the Antenatal School’s offline activities as a platform, the program organized a science communication competition and award ceremony incorporating poetry recitation, music, blessings, and birth dance performances. Participants described this as creating a rich humanistic environment within an artistic ambiance, allowing them to appreciate life, enjoy aesthetic pleasure, cultivate sentiment, experience cultural charm and humanistic beauty, thereby shaping their own aesthetic and value systems.

### 4.3 Perceived integration of “Truth,” “Goodness,” and “Beauty” to enhance comprehensive quality

This study, considering the academic background of third-year nursing students, the characteristics of obstetric nursing, and the needs of pregnant women, built upon students’ existing rational quality. Leveraging the Antenatal School, it extended the teaching venue from the classroom to the antenatal school and labor room, achieving a breakthrough from inside to outside the classroom. Students participated in online lectures and Q&A, engaged in antenatal classes from the pregnant woman’s perspective, and observed real births—activities that participants associated with the pursuit of “Truth.” Through authentic contact with pregnant women and teachers, students reflected on their intellectual engagement, moral sensibility, and interpersonal sensitivity—experiences they linked to the pursuit of “Goodness.” The integration of disciplinary education with art and ideological education, using poetry, birth dance, and blessing rituals to create an artistic atmosphere—was perceived by participants as embodying the pursuit of “Beauty.” Centering students as active participants, the teaching activities appeared to ignite professional passion within authentic contexts, guiding them to appreciate the “beauty of safeguarding life” and achieving an integrated experience of Truth, Goodness, and Beauty to enhance comprehensive quality.

To meet the growing demand for healthcare services, nursing schools must implement national “14th Five-Year Plan” requirements for high-quality development. Cultivating high-level nursing talent is crucial for precisely meeting multi-level, diverse health service needs and expanding nursing service provision. The overarching goal is to train Advanced Practice Nurses (APNs) who “understand medicine, excel in nursing, embody benevolence, and return to the bedside.” Fundamental tasks include fostering virtue, upholding benevolence as the educational tenet, and orienting practice toward clinical competence. Universities should implement systematic humanistic quality education projects to cultivate patriotism and humanistic character, enhancing students’ capacity for humanistic care in clinical nursing, and strengthening the “soft skills” of both students and practitioners (29). Diverse teaching activities motivate student participation and enrich learning experiences. Constructivist theory posits that learners acquire knowledge through situated contexts, collaboration, discourse, and meaning-making (30). Within teaching activities, students are the protagonists. Teachers assume multiple supportive roles: designers of diverse activities, collaborators in activity implementation, participants in dialogic experiences, and facilitators of meaning construction (31).

### 4.4 Limitations of the study

Several limitations of this study should be acknowledged.

First, this study was conducted at a single university and its affiliated hospital. This single-site design may limit the transferability of the findings to other educational or clinical settings, as teaching practices, institutional cultures, and student populations may vary across different contexts.

Second, all interview participants were female, as male students were not enrolled in the course or invited for interviews. Male nursing students may have different experiences and perceptions, and their inclusion could have enriched the findings. Consequently, the generalizability of the findings to male nursing students is restricted.

Third, all participants had completed the full course requirements and voluntarily agreed to be interviewed. This sampling strategy may have introduced a positive selection bias, as students who did not complete all course activities or who had less favorable experiences might hold different perspectives. Therefore, the findings primarily reflect the experiences of engaged and motivated students and may not capture the full range of participants’ perceptions.

Future studies should intentionally include students with diverse levels of engagement and, where possible, explore reasons for non-completion or lower satisfaction. Including male participants and sampling from multiple institutions would also strengthen the generalizability and transferability of findings.

Despite these limitations, this study provides an in-depth qualitative exploration of nursing students’ experiences and perceived educational value of antenatal class blended learning. Within the context of this study, the model appears to offer a promising reference for integrating holistic quality education into nursing curricula. Further research is needed to examine its applicability in other contexts.

## 5 Conclusion

This qualitative study explored the learning experiences and perceived educational value of antenatal classes among third-year nursing students. These students stand at the critical juncture between academia and clinical practice, a period that may be important for igniting professional identity and nurturing humanistic spirit. While possessing foundational theoretical knowledge and skills, they may lack deep emotional and perceptual understanding of life and the profession. The findings suggest that the integrated educational approach combining rational, emotional, and aesthetic education may have supported students’ transition from classroom to clinical roles across cognitive, emotional, and behavioral dimensions. Participants reported a sense of enhanced professional identity and achievement, indicating that the program may serve as a meaningful bridge between academia and clinical practice from their perspective.

Focused on “Perinatal Maternal and Newborn Care,” the online component appeared to open new cognitive windows for participants. Offline courses emphasized communication, practical skills, and observation, which seemed to actively engage students’ senses. The culminating experience was the artistic educational experience of the science communication creation and award ceremony. The course design brought healthcare providers, pregnant women, and students together, leveraging authentic experiences that appeared to engage students’ visual, auditory, olfactory, and tactile senses. This may have facilitated a shift from emotional response to behavioral cognition, potentially helping to solidify professional beliefs emotionally. The integration of sensory and artistic experiences appeared to support students’ ability to view nurse–patient relationships from an aesthetic perspective, which may in turn foster humanistic care spirit and contribute to aesthetic sensibility development.

It should be noted that these findings reflect the subjective perceptions of a single cohort of female students who completed all course requirements. Thus, they may not be generalizable to male students, students with lower engagement, or other educational settings.

## Data Availability

The original contributions presented in the study are included in the article/supplementary material, further inquiries can be directed to the corresponding author.
